# Multiway modeling and analysis in stem cell systems biology

**DOI:** 10.1186/1752-0509-2-63

**Published:** 2008-07-14

**Authors:** Bülent Yener, Evrim Acar, Pheadra Aguis, Kristin Bennett, Scott L Vandenberg, George E Plopper

**Affiliations:** 1 Department of Computer Science, Rensselaer Polytechnic Institute, 110 8th Street, Troy, NY 12180, USA; 2 Department of Biology Rensselaer Polytechnic Institute, 110 8th Street, Troy, NY 12180, USA; 3 Institute of Computer Science, University of Tartu, Liivi 2, Tartu, Estonia; 4 Department of Mathematics Rensselaer Polytechnic Institute, 110 8th Street, Troy, NY 12180, USA; 5 Department of Computer Science, Siena College, 515 Loudon Road, Loudonville, NY 12211, USA

## Abstract

**Background:**

Systems biology refers to multidisciplinary approaches designed to uncover emergent properties of biological systems. Stem cells are an attractive target for this analysis, due to their broad therapeutic potential. A central theme of systems biology is the use of computational modeling to reconstruct complex systems from a wealth of reductionist, molecular data (e.g., gene/protein expression, signal transduction activity, metabolic activity, etc.). A number of deterministic, probabilistic, and statistical learning models are used to understand sophisticated cellular behaviors such as protein expression during cellular differentiation and the activity of signaling networks. However, many of these models are bimodal i.e., they only consider row-column relationships. In contrast, multiway modeling techniques (also known as tensor models) can analyze multimodal data, which capture much more information about complex behaviors such as cell differentiation. In particular, tensors can be very powerful tools for modeling the dynamic activity of biological networks over time. Here, we review the application of systems biology to stem cells and illustrate application of tensor analysis to model collagen-induced osteogenic differentiation of human mesenchymal stem cells.

**Results:**

We applied Tucker1, Tucker3, and Parallel Factor Analysis (PARAFAC) models to identify protein/gene expression patterns during extracellular matrix-induced osteogenic differentiation of human mesenchymal stem cells. In one case, we organized our data into a tensor of type protein/gene locus link × gene ontology category × osteogenic stimulant, and found that our cells expressed two distinct, stimulus-dependent sets of functionally related genes as they underwent osteogenic differentiation. In a second case, we organized DNA microarray data in a three-way tensor of gene IDs × osteogenic stimulus × replicates, and found that application of tensile strain to a collagen I substrate accelerated the osteogenic differentiation induced by a static collagen I substrate.

**Conclusion:**

Our results suggest gene- and protein-level models whereby stem cells undergo transdifferentiation to osteoblasts, and lay the foundation for mechanistic, hypothesis-driven studies. Our analysis methods are applicable to a wide range of stem cell differentiation models.

## Background

### Design optimization of tissue structure and function: a systems biology approach

The *structure/function relationship *dogma is central to understanding how biological systems function. The idea is deceptively simple: understanding the structural organization of biological systems, from massive ecological systems to the shape of a single protein, reveals the function of the system. The concept is powerful enough to have inspired a 200+ year-long effort to describe the components of our biological universe in ever finer detail, beginning with the Linnean taxonomic system of cataloging organisms based on their structural similarities, and culminating with microscale descriptions such as the complete genomes of several organisms, including humans [1]. The *reductionist approach *to biological research has thus reigned supreme for generations, and as a result we now understand how the linear arrangement of nucleotides encodes the linear arrangement of amino acids, how proteins interact to form functional groups such as signal transduction and metabolic pathways, etc.

But at each level of biological organization, we reach a wall- having reduced the complex biological universe to a myriad of minute parts, we encounter new forms of complexity: data overload and the "curse of dimensionality [2]." Simply put, we've taken our biological machines apart but can't put them back together again- our ability to accumulate reductionist data has outstripped our ability to understand it. Thus, we encounter a gap in the structure/function relationship: having accumulated an extraordinary amount of detailed information about biological structures, we can't assemble it in a way that explains the correspondingly complex biological functions these structures perform.

This gap is especially evident at the level of tissues, where most diseases and injuries are manifest. Heart disease and cancer remain the top two causes of death in the United States. One fundamental characteristic of both diseases is *tissue failure*: namely, errors in the structural organization and function of cells in the affected tissues. Likewise, it is estimated that one in six US residents requires medical treatment for an injury each year [3], yet the process of wound healing is so complex it is difficult to accurately predict how quickly most serious wounds will heal [4,5]. Existing models of wound healing rely on clinically relevant, but somewhat superficial, measures of tissue state such as reduction in wound area, linear advancement of wound edge, pain, and ease of use [4-6]. In fact, despite a multitude of genetic screens, biochemical assays, and imaging techniques, the "gold standard" for diagnosis and evaluation remains the expert opinion of highly trained pathologists who scan samples of the tissues in histopathology slides. In other words, the *human eye *is currently the most accurate tool we have available for identifying telltale alterations in the structure and function of diseased and damaged tissues. And it is clear that human judgment is not fail-proof: thousands of diseases are misdiagnosed every year, costing hundreds of millions of dollars in wasted or ineffective medical treatment.

To improve diagnosis and treatment of diseases and wounds, we need a better understanding of how the tremendous numbers of cellular and subcellular parts are organized into functional tissues. One strategy for achieving this is to employ robust methods for describing complex systems, adapted from math and engineering disciplines far outside traditional biomedical fields [7]. Viewed from this perspective, tissue organization and function can be treated as a design optimization problem: what is the optimal arrangement of cellular constituents that achieves the best tissue performance?

When applied to problems of biological complexity, this design optimization approach is sometimes called systems biology. If one begins with the assertion that healthy, native tissues represent a design optimum, the task of systems biology is to identify the "control knobs" that govern tissue structure and function, and the specific "settings" of these knobs that yield an optimally functional (i.e., healthy) tissue. A second important assertion is that tissues are "self-correcting," in that when damaged, they are capable of generating an appropriate response that restores them to their optimal condition (i.e., wound healing): how are the control knobs "turned" to restore optimal function in a wounded tissue? All systems biology approaches therefore focus on defining three characteristics common to self-correcting systems: *robustnes*s (ability to maintain phenotypic stability in response to perturbation), *modularity *(clustering of components into functional "teams"), and, most importantly, *emergent properties *(behaviors unique to the entire system, and not found in any of its constituents)[8]. Systems biology approaches also share a concept known as *iterative refinement*, meaning that they cycle between perturbing a biological system, analyzing the data thus generated, and predicting how the system will respond to a new perturbation; these cycles *learn *relationships.

### Applications of tissue structure and function principles: tissue engineering

Since the late 1980s engineering design principles have been applied to living systems to create replacement tissues de novo [9]. In its most basic sense, an engineered tissue construct (ETC) is a three-dimensional assembly of one or more *cell *types suspended in an extracellular *scaffold *material and fed by *soluble molecules*, including growth factors, hormones, and nutrients [10]. Once assembled, the ETCs are intended to be implanted as replacements for damaged or diseased tissues. The results thus far have been promising [11], and some enjoy widespread use in the clinic [12].

Stem cells are very popular sources for the cellular component in these ETCs because they have the ability to proliferate (thereby populating the ETC with a high density of cells) and undergo differentiation once they reach their desired location and cell density. While embryonic stem cells retain the ability to differentiate into all tissues found in the adult, adult stem cells isolated from a number of different organs retain a more limited differentiation capacity. In either case, the promise is the same: stem cells offer the potential to define and manipulate fundamental principles of cell and tissue behavior, which in turn will uncover a new set of therapeutic targets for correcting errors in cell and tissue function [13].

Due to our very limited understanding of the principles governing tissue structure and function, most current tissue engineering typically follows a trial-and-error approach to design [14]. While persistence can pay off in the long run, the inefficiency of this approach remains one of the major barriers to widespread clinical application of ETCs [15]. Some stem cells used in ETCs also have the capacity to form tumors in vivo[16]. Until it is known how these cells decide which phenotype to adopt, this too presents a significant clinical challenge.

### Human stem cells: attractive targets for systems biology analysis

Systems biology approaches have been employed to help uncover the mechanisms governing differentiation and function of tissue stem cells [17-25] Comparatively little is known about the molecular control of human mesenchymal stem cells (hMSC). Originally described by Friedenstein [26], hMSC are a popular choice for musculoskeletal tissue engineering. hMSC are multipotent, self-renewing cells that can be isolated from the adult bone marrow [27]. They are capable of differentiating into at least three (osteogenic, chondrogenic, adipogenic) and perhaps as many as eight distinct lineages [28].

Due to its complex nature, unraveling stem cell differentiation requires a multi-stage approach. Reductionist studies have thus far identified a small number of potential regulators of this process [29-31], but fail to capture the global effects of these candidates on stem cell behavior. High-throughput, macro-scale studies (genomics, proteomics, etc.) are much better equipped to capture global changes in a complex system, and are the preferred choice for sampling dynamic changes in stem cell "state." But by themselves, these methods are typically not equipped to develop rigorous, testable hypotheses concerning the mechanisms governing this behavior [32].

The second stage of a systems biology approach to cell differentiation is to model the observed protein activity/gene expression changes as a function of the input stimuli. This is where so-called "traditional" biologists collaborate with experts in mathematics and computer science. Several modeling approaches have been applied. For example, Janes and Lauffenburger [33] illustrate how deterministic, probabilistic, and statistical learning models can be used to extract information about proteomic networks. The random nature of proteomic, genomic, and signaling data suggests that machine learning methods can also be used to model complex behavior such as protein-protein interactions [34,35]. For a compherensive review on this topic, we refer the reader to [20,36].

### Application of tensor analysis to stem cell systems biology: multiway models

Faced with analyzing a wealth of data points as inputs, systems biologists turn to dimension reduction techniques using linear algebra. In linear algebra, a matrix is a two-way model used to describe linear relationships between the variables in two dimensions (e.g., rows and columns in a table). For example, in a gene expression experiment, the concentration of a chemical stimulant can serve as the row variables, and the resulting gene expression values can be the column variables. An entry in such a table would describe the gene expression level associated with a particular chemical stimulant concentration. However, it is now quite straightforward to generate data with three or more variables (also known as modes) (e.g., concentration of stimulant, protein phosphorylation level, gene expression level, duration of stimulant exposure, etc.) that cannot be represented by matrices.

**Figure 1:**
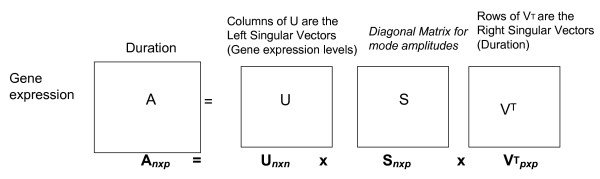
Matrix A is decomposed using singular value decomposition as A = USVT, where U and V are orthogonal matrices containing the left and right singular vectors, respectively and S is a diagonal matrix with the singular values on the diagonal.

Standard two-way dimension reduction techniques such as Singular Value Decomposition (SVD) [37,38] (see Figure 1), which organize data in a matrix form that incorporates time (e.g., concentration of stimulant × duration, protein phosphorylation × duration, or gene expression × duration), identify the linear relationships between modes in a pairwise fashion but cannot analyze three or more modes simultaneously. Uncovering meaningful patterns in a process as complex as cell differentiation, which obviously has far more than two modes, requires a more complex modeling approach.

**Figure 2:**
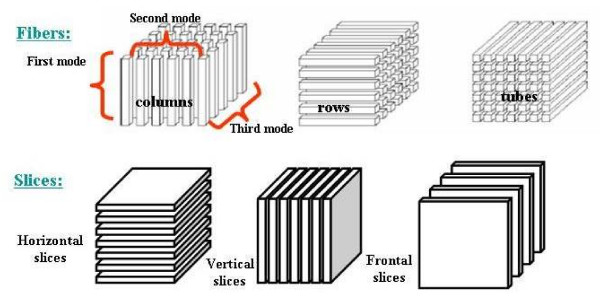
**(Top) FIBERS: (A) Columns, (B) Rows, and (C) Tubes of a 3rd order tensor.** (Bottom) SLICES: (A) Horizontal, (B) Vertical, and (C) Frontal slices of a 3-way tensor (source of the figure is [45]).

This requirement can be satisfied by generalizing matrices to higher order models (e.g., moving from tables to n-dimensional cubes) to discover the multilinear relationships among data in datasets that have more than two different modes (i.e., multimodal data). Tensors are multidimensional arrays (also called n-dimensional cubes) ideally suited for multiway analysis of multimodal data. Figure 2 (top) illustrates the rows (X axis), columns (Y axis) and tubes (Z axis) of a sample of 3-way data array [39] while Figure 2 (bottom) illustrates the "slices" (known as elements) of the cube that correspond to the different modes in the data. In this way, one can generate several different matrices in the 3-way data cube. Though it is difficult to visualize, this approach can also be used to represent four, five, or more different modes simultaneously.

Having generated an "n-dimensional cube" data structure to represent the data, we now turn to the problem of how to fit a multiway model to the data and analyze the multilinear relationships between the modes. Two common models in multiway data analysis are Tucker3 [40-42] and PARAFAC [43].

Both methods model the original data by assembling a substantially smaller dataset representing the larger original data. While PARAFAC has not been applied to tackle the question of cell phenotype changes over time under different stimuli, Omberg et al. did use a Tucker3 (N-mode SVD) approach to model the time course of global gene expression in response to cell cycle inhibitors in yeast [44].

### Overview of multiway modeling and analysis techniques

A higher-order tensor is a multiway dataset represented as T ∈ R^I × J ×....N^, where M > 2. (Note that there are several notations used for representing multiway data sets such that both *T *and $T\in {\text{R}}^{{\text{N}}_{1}\times {\text{N}}_{2}\times ..{\text{N}}_{\text{M}}}$ refer to the same multiway array and will be used interchangeably.) For example consider a 3-way tensor i.e., a data cube that has locus links as its rows, gene ontology categories as its columns, and experimental conditions as its tubes. Below we will discuss three main techniques for analyzing such a tensor.

### Tucker1

**Figure 3:**
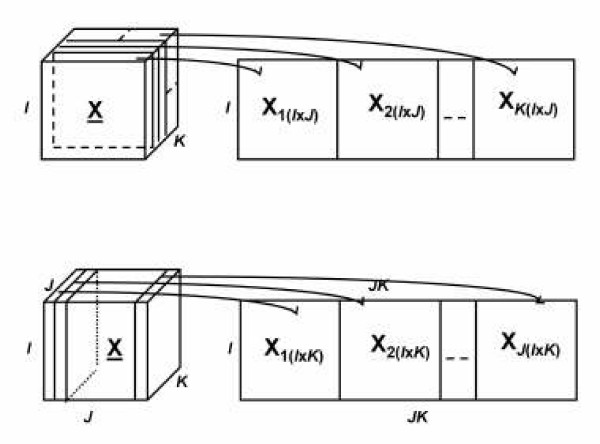
**Unfolding a third order tensor in the first (top) and second (bottom) mode**[50].

Matricizing (unfolding/flattening) rearranges three-way data as a matrix. Thus, it enables us to apply two-way analysis techniques, e.g., SVD, on a three-way dataset. As an example we illustrate matricization of a three-way tensor in the first mode (refer to Figure 3.). In the Tucker1 model the tensor is matricized in the mode of interest and SVD is performed on the corresponding matrix [45].

### Tucker3

**Figure 4:**
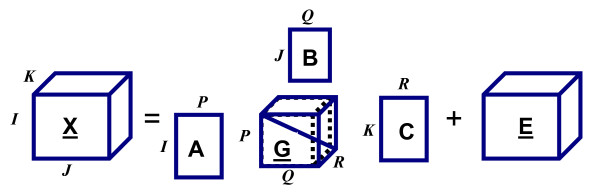
**A Tucker3 Decomposition, where tensor X is decomposed into component matrices A, B, C and core tensor G.** Tensor E contains the error term for each entry in X [33].

One of the most common multiway analysis techniques is the Tucker3 model. As depicted in Figure 4, a 3-way tensor T ∈ R^I × J × K ^is modeled as follows using a Tucker3 model :

$$T_{ijk}=\sum_{\text{r}=1}^{\text{R}}\sum_{\text{q}=1}^{\text{Q}}\sum_{\text{p}=1}^{\text{P}}G_{ijk}{\text{A}}_{\text{ip}}{\text{B}}_{\text{jq}}{\text{C}}_{\text{kr}}+E_{ijk}$$

where P, Q and R indicate the number of components extracted from first, second and third mode (P ≤ I, Q ≤ J and R ≤ K), respectively. A ∈ R^I × P^, B ∈ R^J × Q ^and C ∈ R^K × R ^are the component matrices. *G *∈ R^P × Q × R ^is the core tensor and *E *∈ R^I × J × K ^represents the error term. Different constraints such as nonnegativity, unimodality or orthogonality can be enforced on the component matrices. A Tucker3 model with orthogonality constraints on component matrices is a generalization of SVD from matrices to high-order datasets and is also called Higher-Order Singular Value Decomposition (HOSVD) [46] or multilinear SVD.

Tucker3 is the most flexible model among multiway analysis techniques. Although it suffers from core rotations, which result in non-unique solutions, a Tucker3 model may be preferred over other multiway analysis methods such as PARAFAC because of its flexibility in extracting a different number of components in each mode.

### PARAFAC/CANDECOMP

The simplest three-way model in terms of interpretation is Parallel Factor Analysis (PARAFAC) by Harshman [43] or Canonical Decomposition (CANDECOMP) by Carroll & Chang [47]. These two methods were originally proposed independently, but they employ the same model and are therefore considered completely equivalent.

**Figure 5:**
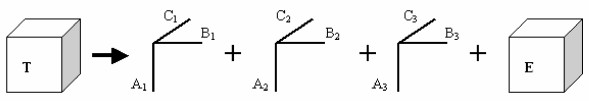
**PARAFAC.** In this example a three-way tensor T is modeled as a sum of three rank-one tensors.

An R-component PARAFAC model on a third-order tensor *T *∈ R^I × J × K ^extracts three component matrices with R factors:

$$T_{ijk}=\sum_{r=1}^{\text{R}}{\text{A}}_{\text{ir}}{\text{B}}_{\text{jr}}{\text{C}}_{\text{kr}}+E_{ijk}$$

where R is the number of components extracted in each mode. A ∈ R^I × R^, B ∈ R^J × R^, and C ∈ R^K × R ^are the component matrices, and E ∈ R^I × J × K ^is the error term. An illustration of PARAFAC decomposition (Figure 5) gives more insight about how the model works. PARAFAC is a restricted Tucker3 model, where the core tensor is superdiagonal, meaning that only the entries G_iii _in core tensor G can be nonzero for all i = 1..R.

The multiway modeling and analysis techniques presented above were applied to two systems biology problems: (i) discovering functional clusters of gene/protein expression during stem cell differentiation, and (ii) dynamics of hMSC osteogenic differentiation over time.

## Methods

In this section we will apply multiway modelling and analysis techniques into two problems. First we will extend our bilinear work in [48] to 3-way modelling and analysis to investigate the impact of different stimulants on hMSC osteogenic differentiation. The main result here is the confirmation of multiple paths for reaching osteoblast. Second, we focus on the time domain and model how osteogenesis evolves as a function of time under tensile strain. Our main finding is that tensile strain accelerates the osteogenic differentiation.

### Case study I

#### Impact of different stimulants on hMSC osteogenic differentiation

In Bennett et al. [48], we examined the protein expression profiles of four populations of hMSC stimulated to undergo osteogenic differentiation via either contact with pro-osteogenic extracellular matrix (ECM) proteins (collagen I, vitronectin, or laminin-5) or addition of osteogenic media supplements (OS media). Unstimulated hMSC and fully differentiated human osteoblasts (hOST) were included as the start and desired end points of this differentiation, respectively. Our goal was to identify key changes in protein (and hence, gene) expression as hMSC move from an undifferentiated state (represented by unstimulated hMSC) to an osteogenic phenotype (represented by hOST). To capture a large amount of protein expression data from each population, we used two-dimensional liquid chromatography tandem mass spectroscopy (2D LC-MS/MS) to detect the 1000 most abundantly expressed proteins in each population of cells. The experiment was repeated three times, yielding three sets of data for each of the cell populations. Our hypothesis was that as hMSC differentiate into the hOST phenotype, their protein/gene expression profile grows increasingly similar to that of hOST. To our surprise, we discovered that, while this appeared to be true, OS and ECM stimulants triggered a rise in different sets of genes found in hOST. These results suggest to us that neither ECM or OS yield a complete osteogenic phenotype when used alone, and that they enhance different types of osteogenic genes. In other words, hMSC exhibited at least two different patterns of protein/gene expression change during osteogenic differentiation. Our results in [48] were mainly obtained by using bilinear methods (although we briefly introduced multiway analysis using Tucker3 method on locus link mode only). Next we show in detail how to fit several multiway models to this multiarray data and analyze its structure.

### Constructing a third order proteomics tensor

In [48] we identified 361 proteins (not expressed by the undifferentiated cells) that varied amongst the hMSC and hOST populations, and annotated the gene for each with its locus link number. We also mapped each gene in a gene ontology tree. The data are arranged as a tensor of type protein/gene locus link (LL) × category (Gene Ontology-GO) × stimulant, with dimensions (361 × 69 × 5), which we call the *Proteomics Tensor *(*T*). The stimulants were numbered as: collagen-I = sample 1, OS = sample 2, vitronectin = sample 3, hOST (positive control) = sample 4, hOST = sample 4, laminin-5 = sample 5.

We applied all three techniques reviewed above to model and analyze the *T*^1^. Tucker1 model had (69, 26, 4) rank reductions for each mode, respectively to explain almost 90% variance of the data. These numbers were used to choose the core component numbers for our Tucker3 model. We also deployed a 12-component PARAFAC model based on the core consistency analysis [50].

### Locus link mode analysis

#### Tucker1 Model

**Figure 6:**
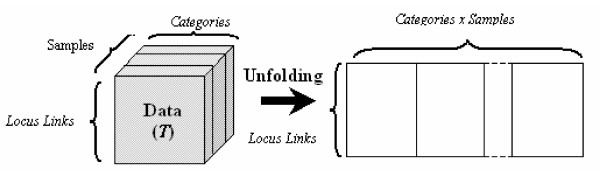
**Unfolding tensor T in the first mode for Tucker1 analysis.** Tensor is reduced to a matrix which permits application of 2-way dimension reduction techniques such as SVD.

We unfold the tensor *T *in the first mode, T_(1) _and apply SVD on T_(1) _to capture the structure in the locus link mode (see Figure 6). Our goal is to use significant left singular vectors to cluster locus links. We select the left singular vectors explaining 90% of total variance, which results in top 69 vectors (in Table 1 we show the first 12 singular values).

**Table 1:** The top 12 singular values of the matrix corresponding to unfolding of tensor T in Locus Link mode indicates that corresponding 12 singular vectors jointly capture only 50% of the original data.

Tucker1: Top 12 Singular (cumulative) Values for Locus Link Mode Matricizing											
0.115	0.178	0.233	0.277	0.318	0.356	0.391	0.422	0.449	0.473	0.496	0.515

Total 243 vectors are needed to obtain 100% explained variance.

#### Tucker3 Model

We choose a model with a core cube that has dimensions 69 × 26 × 4, which explains 82% of the total variance in the data. Note that the choice of the component number combination 69 × 26 × 4 is made based on the rank reductions in the unfolded tensor T and

that there may be other component number combinations that explain the same percent of total variance in the first mode. The core analysis which shows the contribution of each component is given in Table 2.

**Table 2:** Core analysis of the Tucker3 model applied to tensor T shows the contribution of each element in the tensor T to the fitting of Tucker3 model to the data.

ANALYSIS OF 69 × 26 × 4 CORE ARRAY				
Component	Value	Squared	Fraction of Variance	Summed Fraction of Var.
[1, 1, 1]	-21.19	449.10	13.20%	13.20%
[2, 2, 1]	14.77	218.26	6.41%	19.61%
[3, 3, 1]	11.67	136.30	4.01%	23.62%
[4, 4, 1]	10.29	105.94	3.11%	26.73%
[7, 5, 1]	-9.59	91.94	2.70%	29.43%
[6, 1, 2]	-7.71	59.44	1.75%	31.18%
[5, 6, 1]	-7.43	55.14	1.62%	32.80%
[12, 9, 1]	-6.99	48.89	1.44%	34.23%
[3, 1, 3]	6.03	36.37	1.07%	35.30%
[8, 7, 1]	5.94	35.31	1.04%	36.34%
[5, 3, 1]	-5.63	31.71	0.93%	37.27%
[6, 6, 1]	-5.42	29.39	0.86%	38.14%

**Figure 7:**
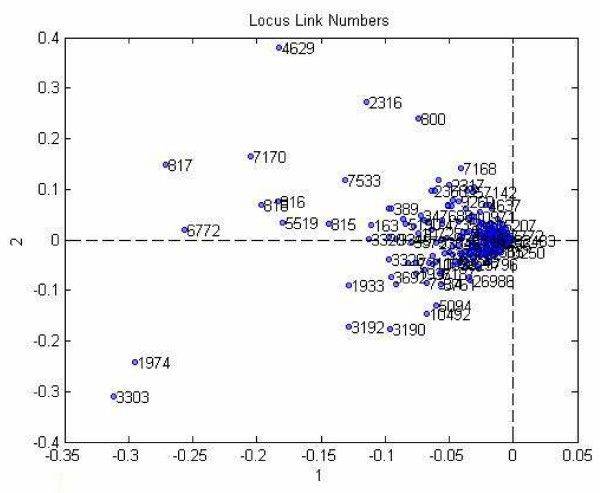
**Projection of the data to lower dimension.** The first two vectors of the locus link component matrix of Tucker3 analysis is chosen as the dimensions.

The projection of locus link numbers onto the first two column vectors of the locus link component matrix is shown in Figure 7. The visual inspection of this scatter plot suggests that the locus link numbers can be clustered into two groups: (i) the outliers, and (ii) the rest. These results are in accord with our previous results reported in Bennett et al. 2007 [48].

#### PARAFAC Model

PARAFAC is a more restricted multiway analysis technique relative to Tucker3, because it requires that (i) the same number of components is extracted from each mode, (ii) a superdiagonal core is constructed. In order to determine the number of components in PARAFAC, we make use of a core consistency diagnostic [50]. As shown in Table 3 as we increase the number of components from 2 to 12 (for component number 1, core consistency is by default 100%), we observe that the explained variance also increases; however, the core consistency values do not exhibit a monotonic decrease. This implies that PARAFAC fails to construct a superdiagonal matrix to fit the data in a consistent manner with the component numbers.

**Table 3:** Core Consistency analysis of PARAFAC model for tensor T.

Comp. #s	1	2	3	4	5	6	7	8	9	10	11	12
Core Con.	100	99.9	99.1	98.1	71.3	82.1	46.2	70.5	76	75	80	82
Expl. Var.	11	17	22.5	25	27	31	34.5	38.1	41.1	43.8	46.1	50.5

The core consistency is given as the percentage of variation in a Tucker3 core array consistent with the theoretical superidentity array. The max value is 100%. A sharp drop in the core consistency value would indicate the number of components to be taken for the modeling [51]. Notice that PARAFAC modeling does not exhibit such a behavior on this data set.

**Figure 8:**
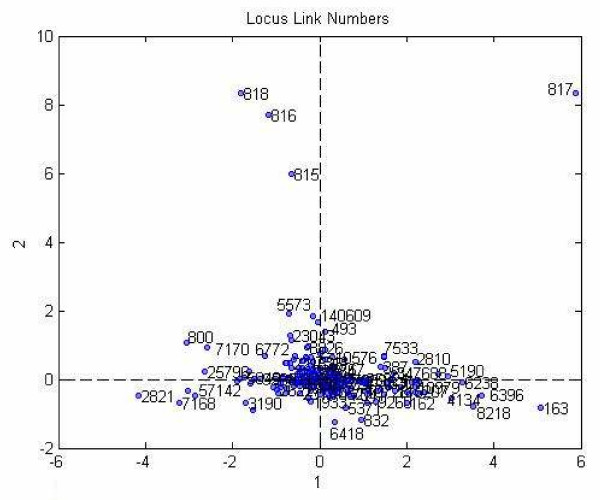
Projection of data into 2 dimensions obtained from the first two component of locus link mode by PARAFAC analysis.

We used the component matrix corresponding to the locus link mode for a 12-factor PARAFAC model to analyze the locus link numbers. A scatter plot of locus link numbers projected on the first two locus link component vectors in shown in Figure 8.

Visual inspection shows that similar to Tucker3 analysis, there is a concentration of a large number of locus link numbers and then there are a small number of outliers.

#### Clustering on locus link mode

We used a k-means clustering algorithm with 100 repeated runs to divide the locus links into two clusters (i.e., k = 2) to identify the outliers from the rest. In order to resolve disagreements between different runs, we computed a majority function by calculating the number of occurrences of a particular clustering and then picking the maximum number of occurrences.

##### Tucker1

**Figure 9:**
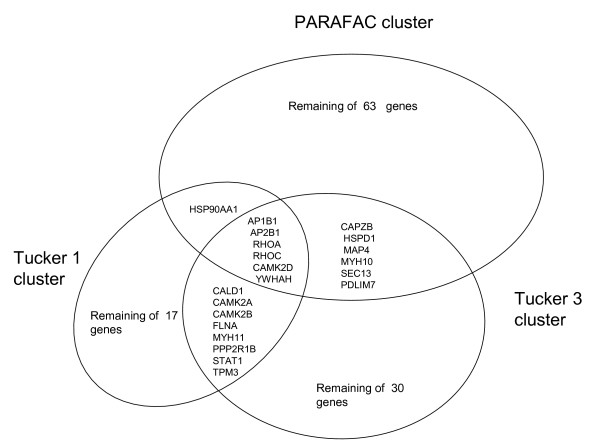
**Venn diagram of k-means clustering results for all three techniques.** Tucker1 and Tucker3 exhibit a stronger agreement than PARAFAC. The detailed interpretation of these clusters can be found at the Discussion section.

First we input the 69 vectors to a k-means algorithm and obtained one cluster with 8 locus link numbers representing the candidate outliers while the rest of the locus links numbers were assigned to the larger cluster. However, k-means algorithm had difficulty to converge on the same cluster assignments for 69 vectors. Thus, we had to cluster in a lower dimension to obtain stability by trading off explained variance: (i) using only the top 12 singular vectors we obtained a cluster with 9 locus links in it that subsumed the first one computed over 69 vectors but clusters were not stable; (ii) inputting only the top 2 singular vectors we obtained very stable clusters that differed contained 17 locus link and subsumed all the elements from the 69 and 12 vector clustering. The summary of k-means algorithms for all three techniques is shown by a Venn diagram in Figure 9.

##### Tucker3

Again, we first input the entire component matrix corresponding to the locus link mode to a k-means algorithm and observed that k-means algorithm on the locus link component matrix with dimensions (361 × 69) had considerable difficulty producing a stable clustering. The maximum occurrence value was close to 40, which is less than 10% of the runs. Thus we chose the top 2 vectors as the input of the k-means algorithm to produce a stable k-means output over 100 runs which constructed a smaller cluster with 30 locus links.

##### PARAFAC

We input the entire component matrix corresponding to the locus link mode for a 12-factor PARAFAC model to cluster locus links. We observed that a k-means algorithm produced more stable clustering results on the locus link component matrix with dimensions (361 × 12) obtained from PARAFAC model. However for consistency we also computed the clustering by using only the top 2 vectors and obtained one small cluster with 63 locus links and one large cluster with the remaining locus links. Comparison of the clusters detected using Tucker1, Tucker3 and PARAFAC methods are given in Figure 9. Notice that while the clusters are not identical across all three methods, there is considerable agreement on the outliers.

### Category mode analysis

The original proteomics data contained 71 gene ontology categories. We elected to drop the categories corresponding to *Transmembrane Receptor Activity *and *Transmembrane Receptor Protein Tyrosine Kinase Signaling Protein *because they contained very few genes. We have analyzed the data by using all three techniques considered in this work. Our analysis in Category mode also aims at identifying clusters of interest that may include categories that are nontrivially related.

**Figure 10:**
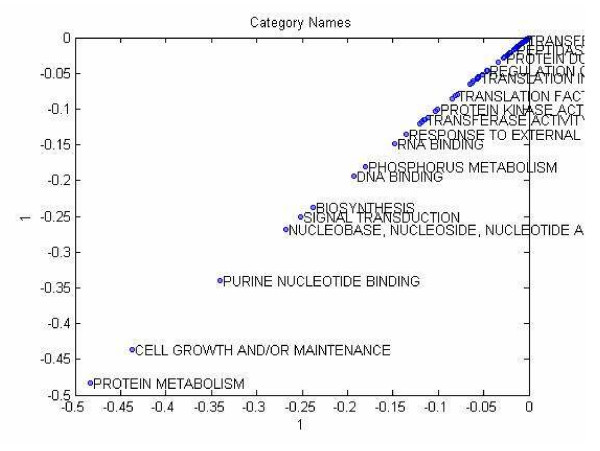
Scatter plot of the category names projected on the 1'st vector of the category component matrix of Tucker3 analysis.

**Figure 11:**
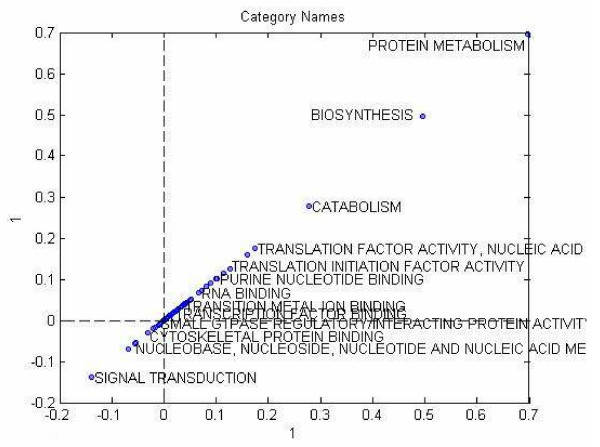
Scatter plot of he category names projected on to the first component of category mode in PARAFAC analysis.

We calculated a scatter plot of category names by projecting them on the first column vector of the category component matrix. The plotting is decluttered to provide a visualization of the clusters as shown in Figure 10. Similar to Tucker3 analysis, we plotted the category names by projecting them on the first column of the component matrix to obtain an intuition about the clustering by visualization as shown in Figure 11.

#### Clustering of gene categories

##### Tucker1

We unfolded the proteomics tensor in the Category mode and computed SVD on the corresponding matrix. 24 singular vectors out of 69 total were sufficient to explain the data with 90% accuracy. These vectors were input to a k-means algorithm for clustering the categories into 4 clusters (i.e., k = 4). We chose four clusters because our previous proteomics analysis identified four classes of differentially expressed proteins/genes between naïve hMSC and osteoblasts [49,51]. The clustering algorithm was run 100 times and the majority calculation was performed as explained in locus link mode analysis above. The Tucker1 method organized the outlying categories into four clusters as shown in Table 4 which we shall interpret at the Discussion section in detail.

**Table 4:** k-means clustering results obtained from 24 singular vectors of the unfolded tensor in category mode.

	Category Names
T1Cluster 1	BIOSYNTHESIS, PROTEIN METABOLISM
T1Cluster 2	DNA BINDING, NUCLEOBASE-NUCLEOSIDE-NUCLEOTIDE AND NUCLEIC ACID METABOLISM, RNA BINDING
T1Cluster 3	CALMODULIN BINDING, CELL GROWTH AND/OR MAINTENANCE, CELL MOTILITY, CYTOSKELETAL PROTEIN BINDING, ORGANOGENESIS, PURINE NUCLEOTIDE BINDING, SIGNAL TRANSDUCTION
T1Cluster 4	The rest of the 69 catagories

##### Tucker3

We input all 24 columns of the category component matrix, which has dimensions (69 × 24), to a k-means (for k = 4) algorithm and ran it up to 100 times to obtain a consensus, or a majority. The clusters and their members are shown in Table 5.

**Table 5:** k-means clustering results obtained from the 24 column vectors of the category component matrix of Tucker3 analysis.

	Category Names
T3Cluster1	BIOSYNTHESIS, CALCIUM ION BINDING, DNA BINDING, PROTEIN METABOLISM, PURINE NUCLEOTIDE BINDING, RNA BINDING
T3Cluster2	CELL GROWTH AND/OR MAINTENANCE
T3Cluster3	CYTOSKELETAL PROTEIN BINDING, SIGNAL TRANSDUCTION
T3Cluster4	The rest of the categories.

The Tucker3 analysis was less informative and the clear distinction between signal transduction and gene expression was lost. Also, the category of calmodulin binding was absent and the category of calcium ion binding, a less specific category, was selected. Other attractive categories in the Tucker1 analysis (signal transduction, purine nucleotide binding, DNA binding) also appeared here, suggesting they may play an especially prominent role during osteogenesis.

##### PARAFAC

We used the component matrix corresponding to the locus link mode for a 12-factor PARAFAC model to cluster locus links using a k-means clustering algorithm for k = 4. The clustering results are shown in Table 6.

**Table 6:** k-means clustering of category names for PARAFAC analysis.

	Category Names
ParCluster1	BIOSYNTHESIS, DNA BINDING, NUCLEOBASE- NUCLEOSIDE-NUCLEOTIDE-AND-NUCLEIC-ACID-METABOLISM, PROTEIN METABOLISM, RNA BINDING
ParCluster2	CELL GROWTH AND/OR MAINTENANCE, CELL MOTILITY, CYTOSKELETAL PROTEIN BINDING, ORGANOGENESIS, PHOSPHORUS METABOLISM, PURINE NUCLEOTIDE BINDING, SIGNAL TRANSDUCTION
ParCluster3	IMMUNE RESPONSE, RESPONSE TO EXTERNAL STIMULUS, RESPONSE TO STRESS
ParCluster4	The rest of the categories

#### Comparison to two-way analysis results

When compared to the categories identified by our previous two-way analysis [48,49], all three tensor models generated more meaningful clusters of functionally related protein categories. Also, many of the categories selected by the tensor models were more specific than those we found previously (e.g., protein metabolism vs. amino acid and derivative metabolism). Curiously, the category of organogenesis, which is perhaps the most directly related to the osteogenic differentiation we are inducing, appeared only in the Tucker1 and PARAFAC models, and was clustered with signal transduction categories in each case. Many of the categories that appeared in the two-way analysis are concerned with activities shared by all cells (e.g., oxidoreductase activity, protein translation, oxygen metabolism).

### Sample mode analysis

#### Tucker1 model

We unfolded the tensor *T *in the third mode (sample mode as shown in Figure 6), T_(3) _and then applied Singular Value Decomposition (SVD) on T_(3) _to capture the structure in sample mode. The largest 4 singular values out of 5 nonzero singular values belong to the singular vectors that explained almost 93% of the variance (1^st^: 53%; 2^nd^: 15%; 3^rd^:14.5%; 4^th^: 10.5).

**Figure 12:**
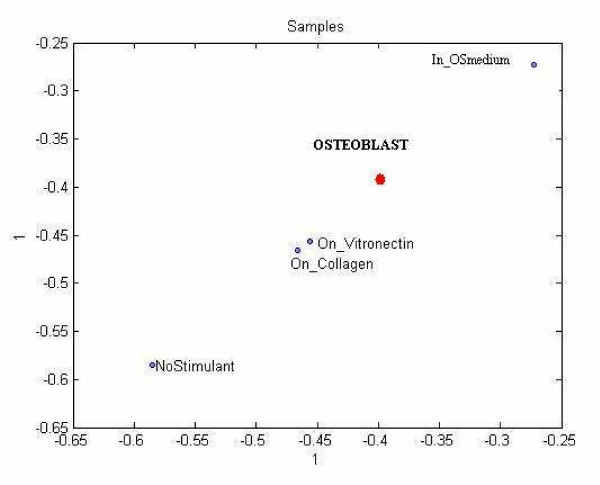
**3D scatter plot of the sample mode entries for Tucker1.** The C1, C2, C2 correspond to the top three component vectors.

Figure 12 shows the scatter plot of the data projected on to the three most significant left singular vectors, which jointly explain almost 90% of the variance.

#### Tucker3 model

We fit our Tucker3 multiway analysis model with a core tensor of dimensions 69 × 24 × 4 to the data tensor, *T *and analyzed the structure in the third mode (sample mode).

**Figure 13:**
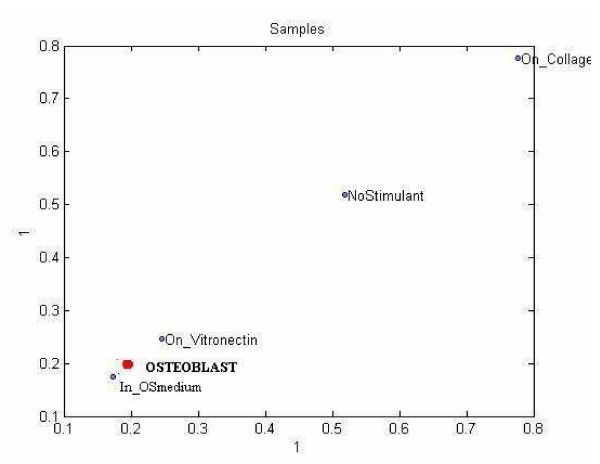
Plotting of sample mode data on to first column of the sample mode component matrix of Tucker3 Model.

Figure 13 shows the projection of the data in samples mode to lower dimension. In particular, we projected the data on to the first column of the sample component matrix to show the scatter plot of the samples. Our interpretation of this plotting is that Osteoblast state can be reached by either In_OSmedium state or by traversing On_Collagen → On_Vitronectin states, which is also reported in [48,49].

#### PARAFAC model

**Figure 14:**
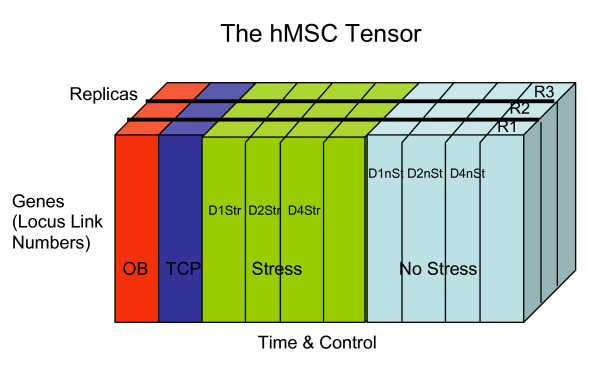
Scatter plot of samples for PARAFAC analysis.

We also decomposed the *T *tensor using a 12-component PARAFAC model and examined the structure in the component matrix corresponding to the sample mode. In Figure 14 we project the data in sample mode on the first component vector of sample mode obtained from PARAFAC decomposition. Interpretation of the scatter plot indicates that PARAFAC modeling does not indicate the existence of different paths toward Osteoblast state.

#### Clustering of sample mode

##### Tucker1

Using the four most significant left singular vectors, we clustered the samples by running the k-means clustering algorithm for 100 times for k = 3. The class assignments for each of 100 runs are stable and shown in Table 7.

**Table 7:** k-means results for all three techniques.

K-Means Cluster Assignment for Samples					
No Stimulant	Collogen	Vitronectin	OS Medium	Osteoblast	

1	1	2	2	3	Tucker1
1	2	1	3	2	Tucker3
1	1	2	2	3	PARAFAC

Again Tucker1 and PARAFAC are in agreement while Tucker3 differs from them.

##### Tucker3

In order to capture the data structure in sample mode, we applied a k-means clustering algorithm on the component matrix corresponding to the third mode. We observed (see Table 7) that class memberships are not the same as in SVD analysis on the unfolded tensor (PT_(3)_) discussed above.

##### PARAFAC

We applied a k-means clustering algorithm to the sample mode component matrix on PARAFAC model which produced the same class assignment as our Tucker1 model, as shown in Table 7.

Clustering based on the PARAFAC decomposition yielded the least informative results, in that the three clusters on the plot formed a pattern lacking any clear, biological meaning.

### Case study II

#### Collagen-induced hMSC stem cell osteogenesis over time

It is clear that cell differentiation is a carefully timed process. What is missing from many systems biology approaches is the element of time, and to add it requires slightly more rigorous analysis. In many cases where data are collected as a function of time, the time element is simply removed, e.g. by taking the maximum activation across the time periods, then using linear two-way causal analysis techniques.

We collected gene expression data (from microarray analysis) for hMSC induced to undergo osteogenic differentiation via two types of stimulus: (1) by simply placing them on a flexible collagen-I coated substrate (unstrained), or (2) by also applying cyclic tensile strain to these substrates. Both conditions were run for five days and triplicate samples collected at day 1, 2, 4 and 5. mRNA from three replicates of naïve hMSC grown on tissue culture plastic (TCP) and fully differentiated hOST were also collected to represent the starting point and desired end point, respectively. The resulting data were filtered as follows: only those genes associated with a locus link number were considered; of these, only those data points tagged as valid (P = present or M = marginal designations) across all 30 samples by the Genespring microarray analysis software were used; of these, only genes with statistically reliable replicates (t-test, 0.05 level of significance) were considered.

Our hypothesis was that application of strain "accelerates" the osteogenic differentiation induced by the collagen I substrate, which will be reflected by the earlier appearance of the osteogenesis-associated genes in the strained samples.

### Constructing a third order time evolving hMSC tensor

**Figure 15:**
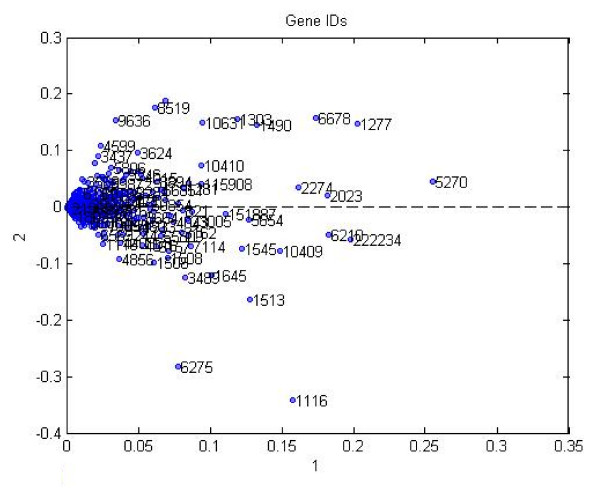
Tensor to model time evolution of stem cell differentiation under different control.

Expressing phenotypic changes over time requires multi-way data analysis, and thus is well suited to tensors. For example, Gaudet's "compendium" [55] includes signaling data triggered by different cytokines at various time points. One tensor in their compendium uses *stimuli, measurements*, and *time *as its modes. Here, we construct a 3-way tensor in which the rows are the gene IDs, the columns are the hMSC populations (negative control cells, hOST, and four samples each of the two stimuli), and the tubes are the replicates (all samples were repeated in triplicate). Over the course of the four time points we measured (days 1, 2, 4 and 5), we detected a total of 3153 genes with statistically significant (t-test, 0.05 confidence level) differential expression, relative to unstimulated hMSC. Thus the hMSC tensor *T *has 3153 × 10 × 3 elements as shown in Figure 15.

**Figure 16:**
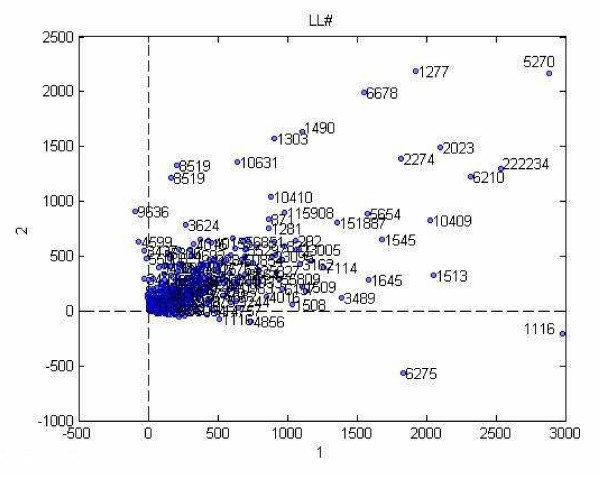
Scatter plot of locus link onto the first vector of the locus link component matrix of Tucker3 analysis.

One objective of our analysis is to understand the evolution of the differentiation process over time, in particular the impact of different stimulants on this process. In Figure 16 we show the scatter plot of the data on the 1st component vector of the time & stimulant (population) mode. The 1st vector separates the two stimuli (static collagen I vs. stretched collagen I) and indicates that adding stretch accelerates osteogenic differentiation toward our target state, represented by fully differentiated hOST. This is consistent with our previous study which focused on a selected set of osteogenic marker genes and demonstrated that application of strain to collagen I induced a more rapid differentiation than static collagen I [28].

#### Tucker3 model

We fit a Tucker3 multiway analysis model with core component numbers 6 × 6 × 3 to the data tensor *T*. The Tucker3 model decomposed the hMSC tensor into three component matrices, one for each mode and a core tensor with dimensions 6*x*6*x*3. The explained variance obtained by this model was 98.16%.

#### PARAFAC model

We analyzed the hMSC tensor with the PARAFAC technique as well and computed the core consistency of our PARAFAC model. Our consistency analysis identified a 2-component model which explained more than 91% of the variance and had 99.71% core consistency (the 3-component model had 37.11% core consistency, which is well below the rule of thumb 90% requirement).

### Locus link analysis

Our objective was to identify the outliers in the locus link mode and examine them in order to learn which genes are potentially important for the cell differentiation process.

For both Tucker3 and PARAFAC we computed the 98% concentration of the locus link numbers projected onto lower dimensions to capture the outliers in the remaining 2 percentile. Finally we took the intersection of the 2 percentile set of Tucker3 and PARAFAC.

### Time analysis

**Figure 17:**
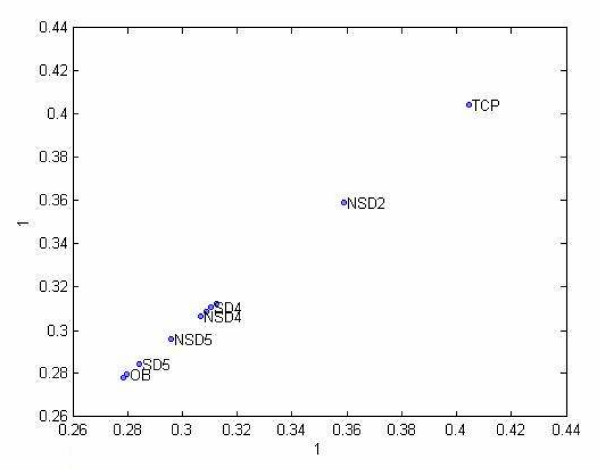
**Time evolution of the hMSC differentiation process.** We projected the data over the first component vector (with explained variance of 85.64%) of the second mode (populations and time) obtained for Tucker3 analysis. At one end we have undifferentiated hMSC (Tissue Culture Plastic, or TCP) and at the other end we have the target state (fully differentiated hOST). In between we plot the data for each stimulus and time point (e.g., NSD2 = no stretch, day 2; SD5 = stretch, day 5). Because the "SD" points lie closer to the target (hOST) than their corresponding "NSD" conditions on days 2 and 5, we conclude that the stimulus "stretch" accelerates osteogenic differentiation when compared to the same stimulus without stretch.

When the data in this mode were projected onto the first component in our Tucker3 model (Figure 17), we observed a striking pattern: fully differentiated osteoblasts and unstimulated hMSC cultured on tissue culture plastic (TCP) lay at opposite ends of the graph, and the hMSC populations cultured on collagen in the presence or absence of mechanical strain were arranged in a temporal sequence, such that cells cultured for two days lie closer to the unstimulated hMSC, and cells cultured for five days lie much closer to osteoblasts.

## Results and discussion

The methods illustrated here provide a means for translating large data sets that capture global gene and protein expression changes during hMSC differentiation into simplified models. The performance of the Tucker1, 3, and PARAFAC models sometimes differed considerably. For example, in our proteomics data set (case study I), Tucker3 appeared to perform best in locus link mode. K-means algorithm identified two clusters (Figure 7), one of which (the outliers) included genes that participate in signal transduction, especially calcium/calmodulin-associated proteins (e.g., calmodulin-dependent protein kinase α, β, and γ); and control of transcription and translation (e.g., STAT1, SYNCRIP). We feel these genes may be of special interest because they fall outside the majority of the "common" genes that can be reduced by the model, and thus may contribute uniquely to the distinct protein profiles in this data set. Tucker1 and PARAFAC, by comparison, were comparatively poor at identifying meaningful clusters. Nevertheless, the outliers identified by each method shared one common feature: they all included a set of six genes, five of which participate in signal transduction pathways (see Figure 10). One of these, calmodulin-dependent protein kinase II delta, was previously identified by SVD analysis as a candidate "osteogenic" gene, in that its expression is found in hMSC populations most closely resembling osteoblasts. This bolsters our belief that calcium/calmodulin signaling in especially important during hMSC osteogenic differentiation.

When one considers the set of proteins shared by at least two of the three methods, this pattern becomes even clearer: additional isoforms of calmodulin-dependent protein kinase II and other signaling proteins (caldesmon, PDLIM7, RhoA, Rho C, and protein phosphatase 2) emphasize the importance of integrin-associated signaling pathways during ECM-induced differentiation. These interpretations are consistent with those of others who have applied similar techniques to other stem cell data sets (refs: UID# 15257023, 17541472, 17625253). These models also included a number of muscle-associated proteins (tropomyosin, two myosin isoforms, CAPZB) suggesting that bone and muscle differentiation may be closely related. This also agrees with our previous analysis of osteogenic gene focusing in response to tensile strain, wherein we observed a drop in expression of marker genes for many different lineages (nerve, fat, cartilage), but observed no drop in smooth muscle cell markers [28].

In category mode, PARAFAC yielded the most interesting clustering results for the proteomics data set, in that it identified clusters of functionally related genes that contribute the most to the model. Furthermore, many of these genes afford a plausible biological explanation for how hMSC undergo differentiation. It selected the greatest number of categories, yet organized them into three clearly distinct clusters. P cluster 1 contained categories primarily concerned with nucleotide binding and metabolism, and resembled the gene expression cluster (T1 cluster 2) in the Tucker1 analysis. P cluster 2 closely resembled the signal transduction cluster (T1 cluster 3) in the Tucker1 model, and added an additional category, phosphorus metabolism. The third cluster contained categories not found in the other two models, that centered on the theme of extracellular matrix protein synthesis and modification. We previously identified these categories as significant during hMSC differentiation [54].

Tucker1 and Tucker3 identified smaller sets of outliers. The first cluster in the Tucker1 model (T1 cluster 1) contained two categories primarily associated with cell survival, and therefore sheds little light on the potential mechanisms underlying hMSC differentiation. However, T1 cluster 2 and T1 cluster 3 contained categories concerned with control of gene expression and signal transduction, respectively. Given the tight association between these activities and their clear association with cellular differentiation, selection of these categories may help identify the potential mechanisms used by hMSC during osteogenic differentiation. In particular, the signal transduction cluster (T1 cluster 3) contained categories concerned with traditional signaling pathways known to control differentiation. For example, calmodulin and calmodulin-dependent protein kinase II stimulate osteogenic differentiation of hMSC while promoting cell migration and suppressing cell growth [52]; all of these activities are contained in the signal transduction cluster. G proteins, which correspond to the purine nucleotide binding category, are well-known to play an important role in osteogenic differentiation [reviewed in [53]]. Again, these results agree with our previous analysis, which identified calcium-dependent signaling as an important factor in osteogenesis [32].

The plot of sample mode data from Tucker3 (Figure 13) is quite informative. The wide separation of the NoStimulant and Osteoblast samples allows us to interpret the space between them as a form of "differentiation axis," and illustrates two important themes. First, we observe that populations of hMSC grown On_Vitronectin or On_Collagen lie midway between the unstimulated hMSC and osteoblasts, demonstrating the partial differentiation induced by these stimulants. Second, the observation that hMSC cultured IN_OSMedium lie beyond the intended target (Osteoblasts) suggests that OS may "over-stimulate" these cells. OS medium contains dexamtheazone, a synthetic form corticosteroid, and this population of cells expressed a distinct set of genes/proteins devoted to steroid metabolism. Both ECM and OS stimulants yield cells that resemble osteoblasts, yet they induce the expression of quite different genes. It is quite possible that the typical OS exposure regimen drives steroid metabolism genes beyond the level necessary for osteogenesis. It is also possible that a combination of the genes expressed in ECM stimulated cells and genes expressed in OS stimulated cells would yield a phenotype closer to true osteoblasts than either set of genes alone. Curiously, the same type of plot for the PARAFAC data (Figure 14) offered no clear biologically meaningful relationship between the samples.

The locus link analysis of our second (microarray) data set identified a set of genes, ("outliers") that our model suggests contribute heavily to the variance between each experimental group (Table 8). In other words, expression of these genes may discriminate between different states of hMSC differentiation. Consistent with our previous analysis, the majority of these genes can be organized into four subsets based on their functions. One class encodes proteins known to contribute to osteogenic differentiation and/or inhibit hMSC growth (FHL2, POSTN, LOX, LOXL1, SPARC, TMSB4X, CTHR1, FST, TGFB1) while a second contains markers for a closely related differentiation fate of hMSC, chondrogenesis (CHI3LI, COL1A1, COL3A1, COL6A3, COL8A1, COL12A1, CTGF, LUM). The fact that many of these are extracellular matrix (ECM) molecules or modifiers of the ECM underscores the importance of ECM in controlling differentiation of hMSC. Expression of at least two of these genes, CTGF and COL12A1, is controlled by mechanical strain. Consistent with our hypothesis that application of strain promotes osteogenic differentiation of hMSC by triggering ECM-associated signaling pathways, our outliers contain a third class of genes that participate in signal transduction and/or regulation of gene expression (IFITM1, CCPG1, FST, MAPKBP1, FBXL2, TRERF1, FHL2). Finally, our gene set contains markers for a range of different cell differentiation fates, including embryonic development (AMD1, SERPINH1), vasculogenesis (S100A4), hematopoesis (B2M, IGFBP6, IKZF5), neurogenesis (BASP1, SERPINH1), and even osteoclastogenesis (CTSB, CTSD, CTSK); it is possible that these genes are downregulated in response to our osteogenic stimulus.

**Table 8:** We intersected the 98% outliers obtained by both Tucker3 and PARAFAC analysis to compose a list of interesting genes

INTERESTING GENES for the DIFFERENTIATION PROCESS:
AMD1 B2M CANX SERPINH1 CHI3L1 COL1A1 COL3A1 COL6A3 COL8A1 COL12A1 COMT CTGF CTSB CTSD CTSK CYP1B1 AKR1C1 ENO1 FHL2 GARS HMOX1 IGFBP6 IMPDH2 LOX LOXL1 LRPAP1 LUM MX1 SERPINE2 HTRA1 RPL27A RPS15A S100A4 SPARC TGFBI TMSB4X UBA1 IFITM1 EIF3D EIF2S2 CCPG1 ISG15 SERF2 BASP1 IFITM3 FST POSTN MAPKBP1 KIF1B FBXL2 C6orf48 TMEM66 CCDC91 TRERF1 C15orf24 IKZF5 BAIAP2L2 DCUN1D5 TUBA1C CTHRC1

Finally, the graph of the samples in Figure 17, when viewed as an axis of similarity between undifferentiated hMSC grown on tissue culture plastic (TCP) and hOST is entirely consistent with our hypothesis, and strongly suggests that hMSC transdifferentiate towards the osteoblast phenotype under these conditions. Furthermore, at each time point tested (days 2, 4, and 5), the strained population always lies closer to the osteoblasts than the unstrained population. This is consistent with our previous finding that application of tensile strain accelerates the osteogenic differentiation of hMSC [31].

## Conclusion

Application of tensor analysis to complex data sets such as those generated in studies of human stem cell differentiation is a powerful method for uncovering important patterns in the data. In particular, we have applied three different analysis methods to two different data sets extracted from hMSC, to yield models that present the data in simplified forms. The first data set was the same one used in [46] while the second one is entirely new.

A cross comparison of the tensor modeling and analysis techniques indicated that the second data set can be modeled and interpreted much better (i.e., by using fewer components, capturing a higher percentage of the variance in the data, and much better consistency in the convergence and fitting). These models also identify candidate genes/proteins as being especially important because they contribute a great deal to explaining the variation between our treatment conditions. It is important that multiple modeling approaches consistently identified a small set of genes that play a large role in differentiating between stem cell populations; these genes thus serve as candidates for hypothesis-driven research aimed at uncovering the molecular mechanisms governing phenotypic changes in stem cells.

While traditional two-way analysis tools are powerful instruments to find relationships in two-way data, the application of tensors allowed us to capture more information than two-dimensional techniques and thus provided a more robust analysis of hMSC differentiation. We feel that our tensor approach has a wide range of possible applications in complex problems in systems biology.

## List of abbreviations

CANDECOMP: Canonical Decomposition; ECM: extracellular matrix; ETC: engineered tissue construct; GO: gene ontology; HOSVD: Higher-Order Singular Value Decomposition; hMSC: human mesenchymal stem cells; hOST: human osteoblasts; LL: locus link; OS: osteogenic supplement; PARAFAC: Parallel Factor Analysis; SVD: singular value decomposition; 2D LC-MS/MS: two-dimensional liquid chromatography tandem mass spectroscopy

## Authors' contributions

BY jointly conceived of the study with GEP and KB, designed the modeling study, and drafted the manuscript. BY and EA performed the tensor modeling and analysis of data. EA helped draft the manuscript. GEP designed the biological experiments, provided biological interpretation of the modelling and analysis of the data, and helped draft the manuscript. KB and SLV acquired and prepared the data. PA filtered and pre-processed the data and helped with post processing of the analysis. All authors read and approved the final manuscript.

## Foot Note

^1^We have used the Matlab with PLSToolbox in our modeling and analysis [49].
